# A Computational Model Predicting Disruption of Blood Vessel Development

**DOI:** 10.1371/journal.pcbi.1002996

**Published:** 2013-04-04

**Authors:** Nicole Kleinstreuer, David Dix, Michael Rountree, Nancy Baker, Nisha Sipes, David Reif, Richard Spencer, Thomas Knudsen

**Affiliations:** 1 National Center for Computational Toxicology, Office of Research and Development, U.S. Environmental Protection Agency, Research Triangle Park, North Carolina, United States of America; 2 Lockheed-Martin, Research Triangle Park, North Carolina, United States of America; University of Virginia, United States of America

## Abstract

Vascular development is a complex process regulated by dynamic biological networks that vary in topology and state across different tissues and developmental stages. Signals regulating *de novo* blood vessel formation (vasculogenesis) and remodeling (angiogenesis) come from a variety of biological pathways linked to endothelial cell (EC) behavior, extracellular matrix (ECM) remodeling and the local generation of chemokines and growth factors. Simulating these interactions at a systems level requires sufficient biological detail about the relevant molecular pathways and associated cellular behaviors, and tractable computational models that offset mathematical and biological complexity. Here, we describe a novel multicellular agent-based model of vasculogenesis using the CompuCell3D (http://www.compucell3d.org/) modeling environment supplemented with semi-automatic knowledgebase creation. The model incorporates vascular endothelial growth factor signals, pro- and anti-angiogenic inflammatory chemokine signals, and the plasminogen activating system of enzymes and proteases linked to ECM interactions, to simulate nascent EC organization, growth and remodeling. The model was shown to recapitulate stereotypical capillary plexus formation and structural emergence of non-coded cellular behaviors, such as a heterologous bridging phenomenon linking endothelial tip cells together during formation of polygonal endothelial cords. Molecular targets in the computational model were mapped to signatures of vascular disruption derived from *in vitro* chemical profiling using the EPA's ToxCast high-throughput screening (HTS) dataset. Simulating the HTS data with the cell-agent based model of vascular development predicted adverse effects of a reference anti-angiogenic thalidomide analog, 5HPP-33, on *in vitro* angiogenesis with respect to both concentration-response and morphological consequences. These findings support the utility of cell agent-based models for simulating a morphogenetic series of events and for the first time demonstrate the applicability of these models for predictive toxicology.

## Author Summary

We built a novel computational model of vascular development that includes multiple cell types responding to growth factor signaling, inflammatory chemokine pathways and extracellular matrix interactions. This model represents the normal biology of capillary plexus formation, both in terms of morphology and emergent behaviors. Based on *in vitro* high-throughput screening data from EPA's ToxCast program, we can simulate chemical exposures that disrupt blood vessel formation. Simulated results of an anti-angiogenic thalidomide compound were highly comparable to results in an endothelial tube formation assay. This model demonstrates the utility of computational approaches for simulating developmental biology and predicting chemical toxicity.

## Introduction

Vascular development is a complex process regulated by biological networks that vary in topology and state across different tissues and gestational stages. Initial stages of blood vessel development in the embryo encompass a morphogenetic series of events from angioblast differentiation into a self-organizing endothelial cell (EC) plexus [1]. This process requires coordinate regulation of complex cellular signals and behaviors such as mitosis, migration, differentiation, adhesion, contractility, apoptosis, and extracellular matrix (ECM) remodeling. A detailed computational model is therefore necessary to understanding both normal embryonic vascular development and how environmental or genetic factors may lead to a variety of developmental defects. Further, due to the significant overlap between developmental and pathological angiogenic signaling [2], such a model could be potentially useful to a wide range of applications in wound healing and tumor angiogenesis, although that is beyond the scope of the current proof-of-concept study.

The cardiovascular system is the first functional organ to develop in the mammalian embryo, reflecting the limits of oxygen diffusion at about 100–200 µm in size (3^rd^ week of gestation in humans, 10^th^ day of gestation in rats, 8^th^ day of gestation in mouse) [1]–[3]. The embryonic vasculature forms through a semi-autonomous process in which EC derived from migratory angioblasts assemble into a primitive multicellular network. This process, vasculogenesis, occurs at different times and locations centrally and peripherally in the embryo and is mediated by cellular processes such as differential migration, proliferation, and adhesion that may form polygonal (roughly hexagonal) whorls of endothelial cords. The endothelial cords undergo tubulogenesis and form a patent system of capillaries that eventually connect into a primitive vascular plexus. Examples include the Perineural Vascular Plexus (PNVP), precursor to the blood-brain barrier, and the peripheral vascular plexus of the limb-bud mesenchyme [4]–[6]. Further growth and remodeling through angiogenesis supports the development of tissues and organ systems through growth and expansion of the primitive vasculature network via sprouting of new capillaries, vessel stabilization and maturation, and flow-based remodeling [7]. Perturbation of embryonic vascular development has the potential to disrupt embryogenesis, leading to adverse pregnancy outcomes such as low birth weight and birth defects [8]. For example, lack of PNVP invasion results in avascular neural tissue, neurodegeneration and embryolethality [9], and inhibition of limb-bud vascularization may contribute directly or indirectly to the origins of phocomelia induced by thalidomide [10], [11]. Analysis of the ToxCast Phase I high-throughput screening (HTS) dataset on 309 environmental compounds, largely pesticides with *in vivo* developmental toxicity information, revealed a strong *in vitro* signature for vascular disruption based upon chemical perturbation of multiple vascular targets and cell systems [12]. The potential molecular targets and key events were further elaborated as an ‘adverse outcome pathway’ framework based on a critical review of literature for embryonic vasculogenesis and angiogenesis [8]. A detailed computational model of critical pathways in vasculogenesis and angiogenesis can thus advance the science closer to predictive understanding of how putative vascular disruptor compounds (pVDCs) might perturb embryonic development [12].

Signals regulating *de novo* blood vessel formation (vasculogenesis) and remodeling (angiogenesis) come from a myriad of biological pathways linked to ECM biology and the local generation of chemokines and growth factors. Vasculogenesis and angiogenesis are regulated by these cell-cell and cell-matrix interactions. These interactions occur between ECs, as well as between EC and other cell types in the microenvironment that control the secretion and release of vascular growth factors and chemokines and contribute to vessel stabilization and sprouting behavior. Heterologous cell types may include inflammatory cells (ICs) such as macrophages and fibroblasts and mural cells (MCs) such as pericytes and vascular smooth muscle cells [2], [5]. Understanding these complex interactions at a systems-level requires sufficient biological detail about the relevant molecular pathways and associated cellular behaviors, and tractable computational models that offset mathematical and biological complexity. The wide range of cell types, signaling molecules and pathways involved in vascular development necessitates a multi-scale modeling approach in which the key molecular pathways and cellular events can be integrated to simulate collective cell behaviors and higher order structure-function.

Agent-based models (ABMs) provide one kind of computational modeling approach that has been used to mathematically describe key cellular events during vasculogenesis and angiogenesis. Previous models have been applied to both developmental and tumor angiogenesis, and have typically focused on one or two cell types (EC, tumor cells), a specific behavior (elongation, ECM interaction, tip cell selection) and the influence of one major growth factor (vascular endothelial growth factor (VEGF)) via either paracrine or autocrine signaling [13]–[18]. Cellular ABMs can simulate discrete cellular behaviors in a multicellular field and solve systems of complex partial differential equations (PDEs) to mimic how cells interact with one another and their microenvironment [19]. In a cellular ABM, cells are represented as agents, i.e., the smallest fundamental units capable of autonomous decisions. Each agent and its interactions are coded into the model based on biological knowledge and experimental data. Emergent properties and phenotypes are then evaluated for biological relevance and insight. One such model utilizing this approach is the Cellular Potts Model, also known as the Glazier-Graner-Hogeweg (GGH) model, implemented in CompuCell3D (CC3D: http://www.CompuCell3D.org) [20]. The GGH method has been extensively validated for multicellular morphogenesis modeling by numerous comparisons between experimental and simulated data for processes in developmental biology including gastrulation, limb-outgrowth, chondrogenesis, angiogenesis, and somitogenesis [16], [21]–[26]. These models link the specific activities of cell signaling pathways to discrete morphogenetic events, and enable hypothesis generation concerning critical developmental driving factors and effects of specific perturbations.

Dynamic cell ABMs have the ability to simulate complex developing systems to a degree of detail that recapitulates tissue-level observations and emergent behaviors [19]. Consequently, there exists the potential to simulate adverse effects that may emerge following exposure to environmental chemicals where there is information on perturbation of the model parameters and signaling networks controlling the simulation. This kind of information can be readily provided via HTS data and *in vitro* assays that are focused on key molecular targets and cellular processes. Here, we present a novel multi-cellular, multi-scale model of capillary plexus formation, implemented in CC3D, and demonstrate the potential application as an *in silico* testing platform for vascular disruption.

## Methods

### Model Topology

The model for embryonic vascular plexus formation was built in CompuCell3D v3.6.0 (http://www.compucell3d.org). We used a two-dimensional (2D) ABM lattice structure based on the observation that lateral splanchnic mesoderm may be represented as a planar surface upon which early embryonic blood vessels form from isolated angioblasts [27]. We therefore neglect critical effects of blood flow that influence arterial/venous specification and shear-based growth or regression later in angiogenic remodeling [28]. We also assume a heterogeneous cell population of endothelial cells (ECs), inflammatory cells (ICs) and mural cells (MCs). As such, we initialized the ABM to represent an approximate distribution of these critical cell types involved in vasculogenesis and angiogenesis in the mammalian embryo and placenta.

### e-Library Construction

An electronic library (e-library) for blood vessel development and remodeling (AngioKB.v1, provided as Supplemental Tables S1–S2) was built and curated semi-automatically from the open scientific literature. Relevant articles were retrieved from PubMed using ChemoText baseline version [29] and the keywords “Neovascularization, Pathologic”, “Neovascularization, Physiologic”, and “Blood Vessels”. These references were then assigned categories based on Medical Subject Headings (MeSH).Those relating to development were parsed out by the following MeSH terms: “Embryonic and Fetal Development”, “Fetus”, “Gene Expression Regulation, Developmental”, “Embryonic Development”, “Growth and Development”, “Embryonic Induction”, “Embryonic Structures”, “[any MeSH heading]/embryology”. This body of literature was then used as a primary source of information to construct the multicellular agent-based model for normal vasculogenesis and for parameter determination. Because our goal was to predict outcome following perturbation, we adopted a general strategy to enable nascent formation of a vascular network by the major cell signals and responses known in embryonic vasculogenesis and angiogenesis (RTK, GPCR and GPI pathways) [8]. Proteins with MeSH terms appearing in these articles were automatically annotated, sorted by occurrence, and cross-referenced with ToxCast assay targets (Table S2). Articles were manually curated to extract relevant cellular biology and parameters such as secretion, diffusion, decay, adhesion, size and motility.

### Mathematical Representations

CC3D represents cells as extended domains of pixels 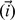 on a lattice, with cell index 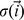 and cell type 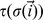. The effective energy, or Hamiltonian (*H_GGH_*), is computed for each cell in the model based on physical features such as volume, membrane area and cell adhesion, as well as dynamic inputs such as the response of a cell to a chemotactic gradient. At every Monte Carlo Step (MCS) or iteration, the effective energy is calculated for each lattice site. The physical inputs into the effective energy are represented in Equations 1–3 (below) where 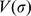 is the cell volume, 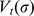 represents the target volume, and 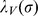 denotes the inverse compressibility (compliance) of the cell. Similarly, 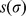 is the surface area, 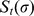 is the target surface area and 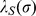 is the inverse membrane compressibility. The target volume and surface area are average values around which the cells will fluctuate based on experimental observations for each cell type, and are shown in Table 1. In IC, MC, and EC-stalk cells, 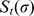 is calculated as 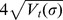. EC-tip cell filopodial extensions are reflected in the higher target surface area (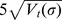) and increased 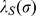 as compared to EC-stalk cells. The presence of cellular adhesion molecules is represented by the boundary energy 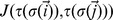 between cell types, shown in Table 2, where a more negative value indicates a higher affinity.

**Table 1:** Physical shape parameter values and equivalent values for each simulated cell type, where *V_t_* represents the target volume, *λ_V_* denotes the inverse compressibility of the cell, *S_t_* is the target surface area and *λ_S_* is the inverse membrane compressibility.

Cell Type	Parameter	Symbol	Parameter Value	Equivalent Value	Ref
EC_t_	Target volume	*V_t_*	30.00	270.00	[55]
	Inverse compressibility	λ_V_	6.00		
	Target surface area	*S_t_*	27.39	82.17	[55]
	Inverse membrane compressibility	λ_S_	8.00		
EC_s_	Target volume	*V_t_*	30.00	270.00	[55]
	Inverse compressibility	λ_V_	6.00		
	Target surface area	*S_t_*	21.91	65.73	[55]
	Inverse membrane compressibility	λ_S_	4.00		
IC	Target volume	*V_t_*	40.00	360.00	[83]
	Inverse compressibility	λ_V_	6.00		
	Target surface area	*S_t_*	25.30	75.90	[83]
	Inverse membrane compressibility	λ_S_	4.00		
MC	Target volume	*V_t_*	50.00	450.00	[32]
	Inverse compressibility	λ_V_	6.00		
	Target surface area	*S_t_*	28.28	84.84	[32]
	Inverse membrane compressibility	λ_S_	4.00		

The simulation was run in 2D, and 1 pixel is assumed to correspond to 3 µm. 2D “volume” units are pix^2^ and µm^2^ and “surface area” units are pix and µm, for parameter and equivalent values, respectively. EC_t_: endothelial tip cells, EC_s_: endothelial stalk cells, IC: inflammatory cells, MC: mural cells.

**Table 2:** Contact energies between cell types (EC_t_: endothelial tip cells, EC_s_: endothelial stalk cells, MC: mural cells, IC: inflammatory cells, ECM: extracellular matrix) are shown, where a more negative number represents higher density of cell adhesion molecules and higher adhesivity.

Adhesion Matrix (Contact Energies: J)							Temperature
	ECM	EC_t_	EC_s_	IC	MC	Apoptotic	(Motility: T)
ECM	0	−4	−4	−4	−4	−2	0
	EC_t_	−7	−8	−5	−4	−2	20
		EC_s_	−8	−2	−6	−2	10
			IC	−2	−2	−3	30
				MC	−4	−2	20
					Apoptotic	0	20

Right column gives the cell-type specific “temperature” value, where a higher value corresponds to increased motility [16], [25].

The dynamic inputs into the effective energy (Equation 4) depend on chemotactic responses to continuously varying concentration fields, solved via reaction-diffusion systems of ordinary and partial differential equations and explained in a subsequent section. The total GGH effective energy (Equation 5) is a summation of the physical and dynamic inputs.
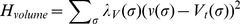
(1)

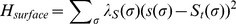
(2)

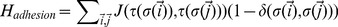
(3)

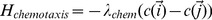
(4)

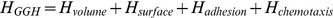
(5)


Cell motility is introduced into the simulation by allowing cells (represented as groups of pixels) from neighboring lattice sites to displace their neighbors (known as an index copy attempt) if it lowers the effective energy. If the effective energy is not reduced the displacement is accepted with a probability, *P*, that decays exponentially in proportion to the effective energy cost, incorporating a stochastic framework into the model known as Metropolis dynamics with Boltzmann acceptance [30]. This is represented in Equation 6, where *θ* is the Heaviside step function and *T(σ)* is the cell-type specific “temperature” value that represents motility (Table 2).
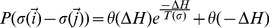
(6)


In concordance with experimental observation, ICs such as macrophages exhibit the most exploratory behavior, followed by EC-tip cells and MCs, with EC-stalk cells being the least motile cell type. Apoptotic cells represent a special case where there is a designated motility value to prevent apoptotic bodies from being static as their volume decreases and they are processed by ICs.

### Cell Types and Signals

The model cell types are endothelial tip cells (EC-tip cells), endothelial stalk cells (EC-stalk cells), inflammatory cells (ICs), mural cells (MCs), and apoptotic cells as well as a representation of the ECM. There are 7 angiogenic signals (VEGF165, VEGF121, CCL2, CXCL10, sVEGFR1, Proteases, and TIE2) that are represented as biochemical fields whose spatial distribution is directed by a system of reaction-diffusion equations overlaid onto the cellular lattice. The remaining 5 angiogenic signals (ANG1, uPAR, PAI-1, VCAM1, and VEGFR2) are represented by their influence on specific cellular behaviors, such as adhesivity, motility and proliferation rate. Table 3 shows the modeling rules applied to the cell types, behaviors, and corresponding angiogenic signals represented in the computational model of early embryonic vascular plexus formation.

**Table 3:** Cell types, behaviors, and associated angiogenic signals represented in the computational model of early embryonic vascular plexus formation.

Cell Type	Behavior	Signal
**Endothelial Tip Cell (EC_t_)**	migration up chemotactic gradients	VEGF165, VEGF121, CCL2
	secretion of proteases that break down ECM and release growth factors	PAI1, Proteases, VEGF165
	expression of chemokines	CCL2
	motility along the ECM	uPAR, VCAM1
	Apoptosis	
**Endothelial Stalk Cell (EC_s_)**	proliferation in response to growth factors	VEGF165, VEGF121
	inhibition of proliferation	CXCL10
	secretion of proteases that break down ECM and release growth factors	PAI1, Proteases, VEGF165
	secretion of soluble decoy receptors that bind and sequester growth factor	sVEGFR1
	adhesion to other cell types	Tie2, VCAM1
	assumption of tip cell type based on free surface area and growth factor concentration	VEGF165, VEGF121
	motility along the ECM	uPAR, VCAM1
	quiescence based on shared surface area with other cells	
	Apoptosis	
**Inflammatory Cell (IC)**	migration up chemotactic gradients	CCL2
	expression of chemokines/growth factors	VEGF121, CCL2, CXCL10
	interaction with the ECM to release bound growth factor	VEGF165
	adhesion to other cell types	VCAM1
	Apoptosis	
**Mural Cell (MC)**	expression of chemokines/growth factors	VEGF165, VEGF121, CCL2
	adhesion to other cell types	ANG1, VCAM1
	motility along the ECM	PAI1
	promotion of endothelial quiescence based on shared surface area	
	Apoptosis	

Signaling molecules diffuse uniformly and isotropically. Lattice boundary conditions are periodic.

ICs are considered to represent generalized macrophage-like cells, as defined by their migratory properties (high motility value) and secretion of inflammatory chemokines [31]. MCs are also represented as generalized cell types that associate with and stabilize the vascular endothelium, including pericytes (e.g., blood-brain barrier) and vascular smooth muscle cells [2], [32]. The ECM is modeled as an additional ‘cell type’ that remains static during the simulation, but can sequester/release growth factors based on protease and IC interaction. Apoptotic cells, regardless of their original cell type, assume a cell type that precedes their breakdown (via incremental decreases in target volume) into apoptotic bodies. There is basal rate of apoptosis represented via cell type switching based on a random number generator whose distribution is tailored to correspond to a 2.5% probability of becoming apoptotic (*P_apop_(σ)*). This was the optimal rate of apoptosis derived from parameter sweeps between 0–25% probability of apoptosis in balancing cell density and overall VEGF concentration. Cytotoxicity may be represented in the model as an increase in the probability of apoptosis for a particular cell type. A basal rate of proliferation is implemented for each cell type, modeled as an incremental increase in the target volume of the cell 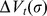. A cell reaching its doubling volume then undergoes mitosis into two daughter cells of the same cell type as the parent. In the case of ECs, there is a VEGF concentration threshold *C_v:thr_* that must be exceeded to stimulate cell growth. Conversely, there is a CXCL10 concentration *C_Cx:thr_* threshold that, once exceeded, counteracts VEGF-stimulated proliferation.

ECs occur as either EC-tip cells or EC-stalk cells, defined in the model as separate cell types. The former are characterized by migratory behavior (higher motility value and chemotactic strengths), exploratory filopodial extensions (higher 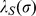) and minimal potential for proliferation, whereas the latter are non-exploratory and proliferative (increase in target volume in response to VEGF) [33]. Each cell in the computational model is able to keep track of its' shared surface area with other cells, and this data is used as an input to certain cellular behaviors. For example, EC-stalk cells adjacent to EC-tip cells express higher levels of soluble VEGFR1 (sVEGFR1), the decoy receptor that sequesters VEGF, creates a ligand corridor for vessel sprouting, and buffers the angiogenic response [34], [35]. Similar to what has been observed experimentally, model ECs are able to switch cell type (tip vs. stalk) based on their local environment. An EC-stalk cell that has <80% shared surface ares with other cells may switch type and become an EC-tip cell based on exceeding a VEGF concentration threshold, leading to potential angiogenic sprout formation. An EC that shares >50% of its' surface area with an EC-tip cell, or is surrounded by other ECs (no free surface area), cannot assume the EC-tip cell type [36], [37]. These rules attempt to mimic the effects of Delta-Notch signaling without an explicit mathematical representation of the intra-cellular molecular networks.

All cells in the model may differentially interact with the full range of signals, detailed in Table 3, through a combination of mechanisms including receptor expression, secretion, chemotaxis, and uptake. VEGF is represented in the model as a freely diffusible isoform (VEGF121) and as an isoform that contains a heparin sulfate binding domain (VEGF165). The latter isoform is liberated from the ECM by proteases secreted by ECs, and by ICs and MCs that interact with and break down the ECM. Sprouting EC-tip cells express higher levels of proteases such as MMP7 than quiescent EC-stalk cells [38]. Endothelial chemotaxis in response to VEGF gradients occurs via VEGFR2-mediated downstream activation of pathways leading to formation of filopodia, stress fibers, lamellipodia and focal adhesion complexes that facilitate migration along the ECM [39]. This is represented in the model by a higher chemotactic strength for EC-tip cells in response to a VEGF gradient. Previous CC3D models of early vascular development have assumed either exclusively paracrine [15] or autocrine [16] VEGF signaling as the driving factor for patterning. Here, we propose both autocrine and paracrine contributions to VEGF signaling whereby soluble VEGF121 is produced by EC-stalk cells, MCs and ICs, and bound VEGF165 is secreted by MCs and liberated from the ECM by ICs and EC-tip cell proteases. This scenario is supported by experimental evidence for cell-specific secretion of VEGF isoforms and by a demonstrated necessity for both the heparin-bound form and the freely diffusible form for correct spatial patterning [6], [32], [40]–[43].

The chemokine CCL2 is expressed at varying levels by all active cell types in the model and causes chemotaxis in EC-tip cells and ICs, and a mitogenic response in MCs [44], [45]. An IC-MC interaction has been shown to synergistically amplify CCL2 production [46], which is thus represented in the model as an increase in CCL2 secretion on contact between the two cell types. In addition to induction of a variety of angiogenic growth factors, receptors and adhesion molecules, CCL2 is also thought to enhance EC responsiveness to VEGF [47]. The anti-angiogenic chemokine CXCL10 is expressed by ICs and inhibits EC-stalk cell proliferation via a competitive mechanism with the heparin-binding site on VEGF165, as well as via CXCR3-mediated apoptosis at higher concentrations [48], [49]. This activity is represented in the model via a threshold-based approach where a shift in the relative concentrations of CXCL10 and VEGF165 causes first a decrease in incremental EC-stalk cell growth and subsequently an increase in EC-stalk cell apoptosis. Angiopoietin-1 (ANG1), a ligand for TIE2 expressed on the MC surface, binds its cognate receptor on EC-stalk cells, represented computationally via the boundary energy between these cell types. The association between MC and EC-stalk cells provides structural support for the nascent vessels, guidance cues from MC-derived growth factors, and promotes endothelial quiescence [2]. The latter effect is represented in the model by contact inhibition of EC-stalk cell proliferation based on shared surface area with MCs.

The effects of the plasminogen activating system (PAS) are represented partly by the aforementioned proteases and partly by expression of the urokinase-type plasminogen receptor (uPAR) and Plasminogen Activator Inhibitor 1 (PAI-1). Through interaction with vitronectin (VN), uPAR influences EC motility and migration along an ECM substratum [50]. PAI-1 has been shown to control MC motility by a similar VN-dependent mechanism [51], [52]. PAI-1 also regulates protease secretion and thus may influence the proteolytic balance of the system. Finally, cell surface expression of the vascular cell adhesion molecule 1 (VCAM1) by ECs, MCs, and ICs [53], [54] is reflected in the model by differing contact energies between cell types.

### Biochemical Fields and Chemotaxis

As shown in Equation 4, chemotaxis is implemented as an energy bias in the direction of higher concentrations, allowing cells to preferentially move up or down a gradient based on the chemotactic field strength *λ_chem_* (Table 4). The majority of the fields (VEGF121, CCL2, CXCL10, sVEGFR1, Proteases, and TIE2) evolve according to the diffusion equation:
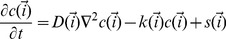
(7)


**Table 4:** Dynamic simulation parameters affecting concentration fields and chemotactic responses.

Molecule	Parameter	Cell/Field Type	Symbol	Parameter Value	Equivalent Value	Ref
VEGF165	Diffusion	ECM	*D_V_*	4.0×10^−2^	0.36	[15], [25], [41], [84]
	Decay	ECM	*k_V_*	1.0×10^−3^	1.0×10^−3^	
	Secretion	IC	*S_V:IC_*	6.0×10^−3^	6.67×10^−4^	[85]
		MC	*S_V:MC_*	5.0×10^−3^	5.55×10^−4^	[32]
	Uptake	EC_t_	*S_V:Ect_*	−2.0×10^−3^	−2.22×10^−4^	
		EC_s_	*S_V:Ecs_*	−2.0×10^−3^	−2.22×10^−4^	
	Field coupling	sVEGFR1	*n_r_*	−0.02		[34]
		Proteases	*n_p_*	0.02		[86]
	Chemotactic strength	EC_t_	λ*_c:V:ECt_*	600		[43]
		EC_s_	λ*_c:V:Ecs_*	300		[43]
VEGF121	Diffusion	ECM	*D_Vf_*	0.18	1.62	[15], [25], [41], [84]
	Decay	ECM	*k_Vf_*	1.0×10^−3^	1.0×10^−3^	
	Secretion	EC_s_	*S_Vf:Ecs_*	2.0×10^−3^	2.22×10^−4^	
		IC	*S_Vf:IC_*	5.0×10^−3^	5.55×10^−4^	[85]
		MC	*S_Vf:MC_*	5.0×10^−3^	5.55×10^−4^	[32]
	(on contact)	EC_s_:MC	*S_Vf:Ecs:MC_*	4.0×10^−3^	4.44×10^−4^	[32]
	Uptake	EC_t_	*S_Vf:Ect_*	−2.0×10^−3^	−2.22×10^−4^	
		EC_s_	*S_Vf:Ecs_*	−2.0×10^−3^	−2.22×10^−4^	
	Chemotactic strength	EC_t_	λ*_c:Vf:ECt_*	400		[43]
		EC_s_	λ*_c:Vf:Ecs_*	200		[43]
CCL2	Diffusion	ECM	*D_C_*	8.0×10^−2^	0.72	
	Decay	ECM	*k_C_*	2×10^−3^	2×10^−3^	
	Secretion	EC_t_	*S_C:Ect_*	4.0×10^−3^	4.44×10^−4^	[87], [88]
		EC_s_	*S_C:Ecs_*	2.0×10^−3^	2.22×10^−4^	[87], [88]
		IC	*S_C:IC_*	6.0×10^−3^	6.67×10^−4^	[87]
	(on contact)	MC:IC	*S_C:MC:IC_*	1.0×10^−3^	1.11×10^−4^	[46]
	(on contact)	IC:MC	*S_C:IC:MC_*	1.0×10^−3^	1.11×10^−4^	[46]
	Chemotactic strength	EC_t_	λ*_c:C:ECt_*	400		[39], [45]
		EC_s_	λ*_c:C:Ecs_*	200		[39], [45]
		IC	λ*_c:C:IC_*	400		[39], [45]
CXCL10	Diffusion	ECM	*D_Cx_*	8.0×10^−2^	0.72	
	Decay	ECM	*k_Cx_*	2×10^−3^	2×10^−3^	
	Secretion	IC	*S_Cx:IC_*	5.0×10^−3^	5.55×10^−4^	[87]
		MC	*S_Cx:MC_*	3.0×10^−3^	3.33×10^−4^	[87]
sVEGFR1	Diffusion	ECM	*D_r_*	0.14	1.26	
	Decay	ECM	*k_r_*	4×10^−3^	4×10^−3^	
	Secretion	EC_s_	*S_r:Ecs_*	2.0×10^−3^	2.22×10^−4^	[38]
	Chemotactic strength	EC_t_	λ*_c:r:ECt_*	−200		[34]
Proteases	Diffusion	ECM	*D_p_*	0.2	1.8	
	Decay	ECM	*k_p_*	5×10^−3^	5×10^−3^	
	Secretion	EC_t_	*S_p:Ect_*	1.0×10^−2^	1.11×10^−3^	[89]
		EC_s_	*S_p:Ecs_*	1.0×10^−3^	1.11×10^−4^	[89]
	(on contact)	EC_t_:IC	*S_p:Ect:IC_*	1.0×10^−2^	1.11×10^−3^	[31], [38]
	Chemotactic strength	IC	λ*_c:r:IC_*	−200		
TIE2	Diffusion	ECM	*D_T_*	0.1	0.9	
	Decay	ECM	*k_T_*	5×10^−3^	5×10^−3^	
	Secretion	EC_t_	*S_T:Ect_*	5.0×10^−3^	5.55×10^−4^	[90]
		EC_s_	*S_T:Ecs_*	1.0×10^−3^	1.11×10^−4^	[90]
	Chemotactic strength	MC	λ*_c:T:MC_*	100		[35]

Diffusion units are pix^2^/s and µm^2^/s, decay units are MCS^−1^ and s^−1^, and secretion and uptake units are c.u./pix^2^/s and c.u./µm^2^/s, for parameter and equivalent values, respectively (c.u. is arbitrary concentration units). Parameter values were informed by experimental observations and in most cases estimated to achieve relative steady state concentrations that approximated measured serum levels. References correspond to measured values (diffusion, secretion) or observed behaviors (field coupling, chemotactic strength). EC_t_: endothelial tip cells, EC_s_: endothelial stalk cells, IC: inflammatory cells, MC: mural cells, ECM: extracellular matrix.

In Equation 7, 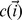 is the field concentration, 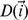 is the diffusion constant, 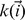 is the decay constant, and 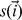 is the secretion rate. In the case of the cell-surface receptor TIE2, the decay rate is greater than or equal to the secretion rate for each EC type, maximizing expression on the cell membrane and minimizing it elsewhere in the ABM lattice. The secretion rate can either assume a constant value for each relevant cell type (sVEGFR1, TIE2, CXCL10) or it may include an additional dependence on contact between specific cell types (VEGF121, CCL2, Proteases). Uptake of the growth factor VEGF121 and VEGF165 by EC-tip cells and EC-stalk cells is represented as a negative contribution to the secretion rate. To solve the concentration field for VEGF165 (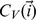 in Equation 8), there are additional terms in the diffusion equation representing field coupling.
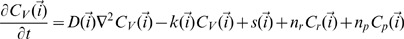
(8)


The terms *n_r_* and *n_p_* denote coupling coefficients for the sVEGFR1 concentration 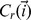 and the protease concentration 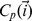. The field coupling with sVEGFR1 is in the negative direction, representing growth factor sequestration, and the coupling with proteases is in the positive direction, representing breakdown of the ECM and release of bound VEGF165.

### Simulation Parameters

The initial configuration of our simulation is a heterogeneously seeded cell field on a hexagonal lattice of 200×200 pixels with periodic boundary conditions. There is a random seed number that ensures that the spatial distribution of the cells varies among simulations; however, the relative percentage of cell types is constant at the start of each run. The starting configuration (∼700 cells/mm^2^) and relative cell densities were estimated based on experimental observation, where ECs constitute 75% (divided between 62.5% EC-stalk and 12.5% EC-tip cells) and the remaining 25% are evenly divided between ICs and MCs [2], [15], [55]. Each pixel corresponds to ∼3 µm in biological space, and each MCS corresponds to ∼1 sec real time. All secretion/diffusion/uptake parameters are unique to each specific concentration field and were informed by the open scientific literature (Table 4). Parameters were converted from measured experimental values, as in the case of diffusion and secretion, or estimated based on observed behaviors as in the case of field coupling and chemotactic strength. When specific experimental values were not available, parameter values were estimated to achieve relative steady state concentrations that approximate measured serum levels in humans. The total simulated time over 10,000 MCS equates to roughly 3 hrs, which agrees with the estimated time scale over which the primitive embryonic capillary plexus forms in mammals *in vivo* [1].

### ToxCast HTS Data

ToxCast Phase I profiled the biological activities of 309 unique chemicals using over 600 HTS assays, including biochemical assays (e.g., nuclear receptor binding, enzyme inhibition), cell-based assays (e.g., cytotoxicity profiles, reporter gene assays), complex culture systems (e.g., embryonic stem cell differentiation, inflammatory/angiogenic signals), and chemical property information. The public *in vitro* dataset and relevant publications can be accessed at http://actor.epa.gov/actor/faces/ToxCastDB/Home.jsp and http://actor.epa.gov/toxrefdb/faces/Home.jsp. Several technology platforms in the ToxCast assay portfolio tested for chemical effects on molecular targets that map to key signaling pathways in vascular development [8], [12]. The reference anti-angiogenic compound, 5-Hydroxy-2-(2,6-diisopropylphenyl)-1H-isoindole-1,3-dione (5HPP-33, ≥98%, Sigma-Aldrich, St. Louis, MO) was identified in a previous structural development study on thalidomide analogs to identify anti-angiogenic compounds in a human umbilical vein endothelial cell (HUVEC) assay [56]. For the present study, this compound was tested in the following HTS assays as part of ToxCast Phase II (>800 chemicals, >650 assays). The full ToxCast Phase II dataset will be available in mid-2013 (http://www.epa.gov/ncct/toxcast/).

#### Attagene platform

This is a cellular biosensor system for rapid, high-content assessment of a compound's impact on gene regulatory networks, with combined libraries of *cis-* and *trans-*regulated transcription factor reporter constructs and a homogenous detection method enabling simultaneous evaluation of multiplexed transcription factor activities [57]. We evaluated the effects of 5HPP-33 on 25 nuclear receptors (TRANS) and 48 transcription factor response elements (CIS), with 10 corresponding receptor family targets between the CIS and TRANS assays.

#### BioMAP platform

This assay system, developed by BioSeek, models complex human disease and tissue biology with co-cultures of primary human cells under various stimulatory conditions [58]. Cell types consisted of HUVECs, fibroblasts, bronchial epithelial cells, smooth muscle cells, keratinocytes and peripheral blood mononuclear cells in 8 different culture conditions. Cells were stimulated with a variety of important biological effectors such as TNF-α, LPS, IL-1β, IFN-γ, EGF, TGF-β and increased or decreased levels of downstream response proteins determined by enzyme-linked immunosorbent assay. Measures of cytotoxicity and proliferation are also used. The effects of 5HPP-33 on a total of 87 BioMAP endpoints are reported here.

#### NovaScreen platform

This biochemical HTS assay portfolio (Perkin-Elmer) was used to test 5HPP-33 in: 77 G-protein coupled receptor (GPCR) binding assays; 10 CYP450-related enzyme activities; enzymatic assays for 37 kinases, 19 phosphatases, 15 proteases, 2 histone deactylases, 3 cholinesterases and 16 other enzyme activities; 19 nuclear receptor binding assays; 20 ion channel and ligand-gated ion channel activities; 9 transporter proteins, 2 mitochondrial pore proteins and 2 other receptor types for a total of 231 assay features described in [59].

The results of each assay with a corresponding molecular or cellular target in vascular development were used to parameterize the chemical-exposure model. Embryonic vascular exposure to 5HPP-33 was simulated using the lowest effective concentration (LEC) values where a statistically significant difference from control was observed. These values were in the micromolar range, and the responses were measured primarily in fold-change of protein expression. The concentration response data (Supplemental Table S3) were used to simulate chemical exposure during embryonic vascular plexus development at low (∼3–5 µM) and high (∼30–40 µM) test concentrations. Where possible, fold changes in protein levels from the concentration response curves were translated directly into parameter fold changes. For example, a 2-fold decrease in the expression of a chemokine in a BioMAP human primary cell system resulted in a 2-fold decrease in the secretion rate parameter for the corresponding cell type and concentration field. Changes in proliferation were equivalently applied as adjustments to the change in target volume of the respective cell types. Other parameter perturbations were more complex, such as expression of surface adhesion molecules which influence cell-cell adhesivity and must therefore be translated into contact energies between cells, a dimensionless computational parameter. Another example is receptor expression influencing cell type-specific motility. In such cases it was not possible to directly convert fold changes so a heuristic was applied in the form of incremental decreases in computational parameters. The specific parameters adjusted to simulate 5HPP-33 exposure, corresponding to ToxCast assay perturbations, are detailed in the Results. Publication and release of the entire ToxCast Phase II dataset, including the subset of data for 5HPP-33 reported here, is scheduled for early 2013. Quantitative analysis of simulated vascular networks was performed using AngioTool (v0.5a, [60]), and statistical analysis was done in R (v2.13.0).

## Results

### Normal Vascular Development (Control Model)

**Figure 1:**
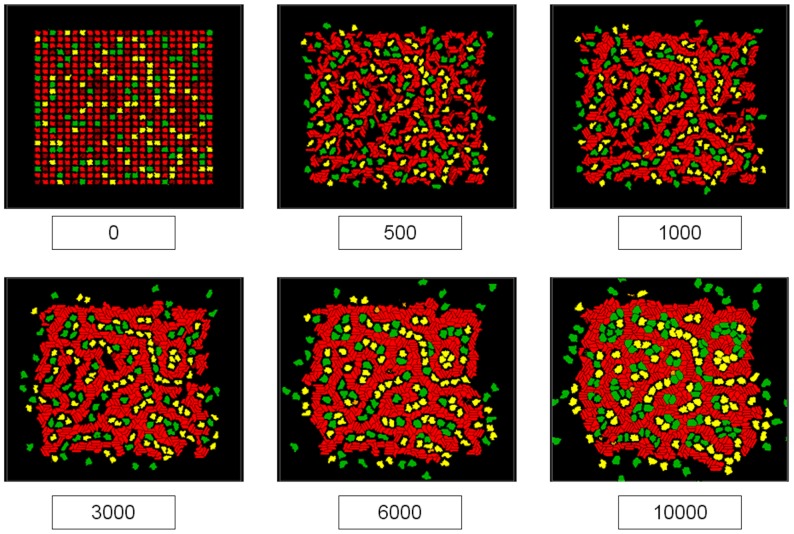
Control model of early embryonic vascular plexus formation. Labels represent simulation time in MCS, where the simulation was run for 10,000 MCS (∼3 hrs). Frames display the progression of interactions between endothelial cells (red), mural cells (green), and inflammatory cells (yellow) as a stable polygonal plexus of endothelial cords emerges. The movie file for the control model is provided as Supplemental Video S1.

The cellular ABM was run for 10,000 MCS to simulate the early stages of embryonic vascular plexus formation. Figure 1 displays the progression of the model as the simulation advances over six time points. The model can be viewed as a 2D cross section of a nascent capillary plexus comprising ECs (Figure 1, red cells) supported by MC (green cells) and IC (yellow cells). ECs initially elongate due to chemotactic forces leading to the formation of vascular cords. Under the mitotic influence of growth factors liberated from the ECM and secreted by the other cell types, EC-stalk cells proliferate while EC-tip cells extend exploratory filopodia into the microenvironment. This results in a rudimentary capillary plexus co-opting all ECs into the network by approximately 3000 MCS. At this stage, the model has achieved a steady state in which the overall distribution of cell types and general topology of the cellular network does not change significantly throughout the remainder of the simulation. However, the vascular network remains dynamic through 10,000 MCS and continues remodeling via random apoptosis as well as complex cell signal-response interactions leading to proliferative vessel thickening and stabilization by MC adhesion. As such, the capillary network is not programmed *a priori* but rather emerges from the system of complex interactions enabled by heterogeneous cellular properties and local environmental signals.

**Figure 2:**
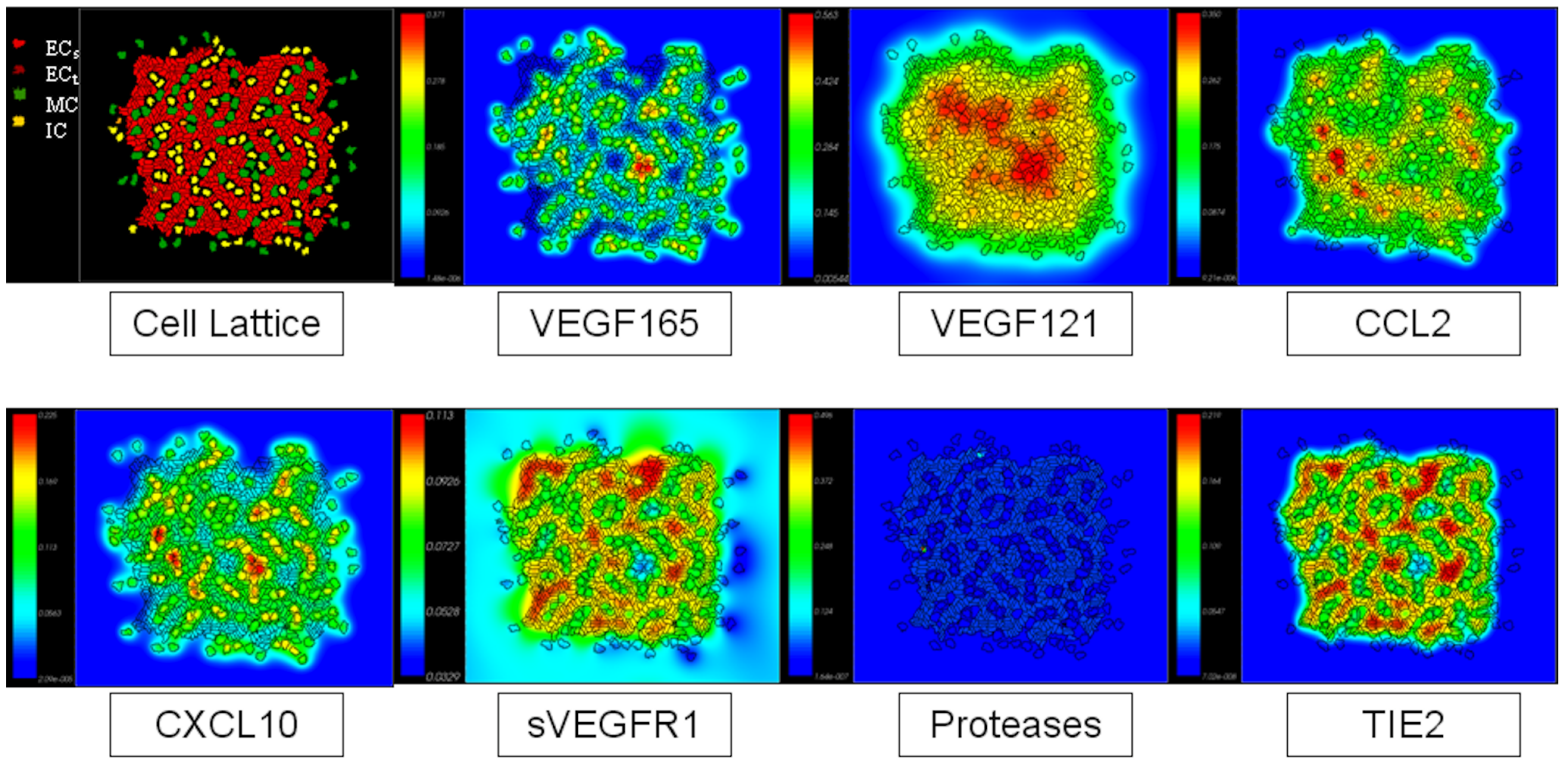
Control model of early embryonic vascular plexus formation at ∼5000 MCS, showing the cellular lattice and overlaid molecular signaling concentration fields. At this stage, there are low levels of protease expression though it can be seen around the sprouting tip cells. The movie file for the control model with overlaid concentration fields is provided as Supplemental Video S2.

**Figure 3:**
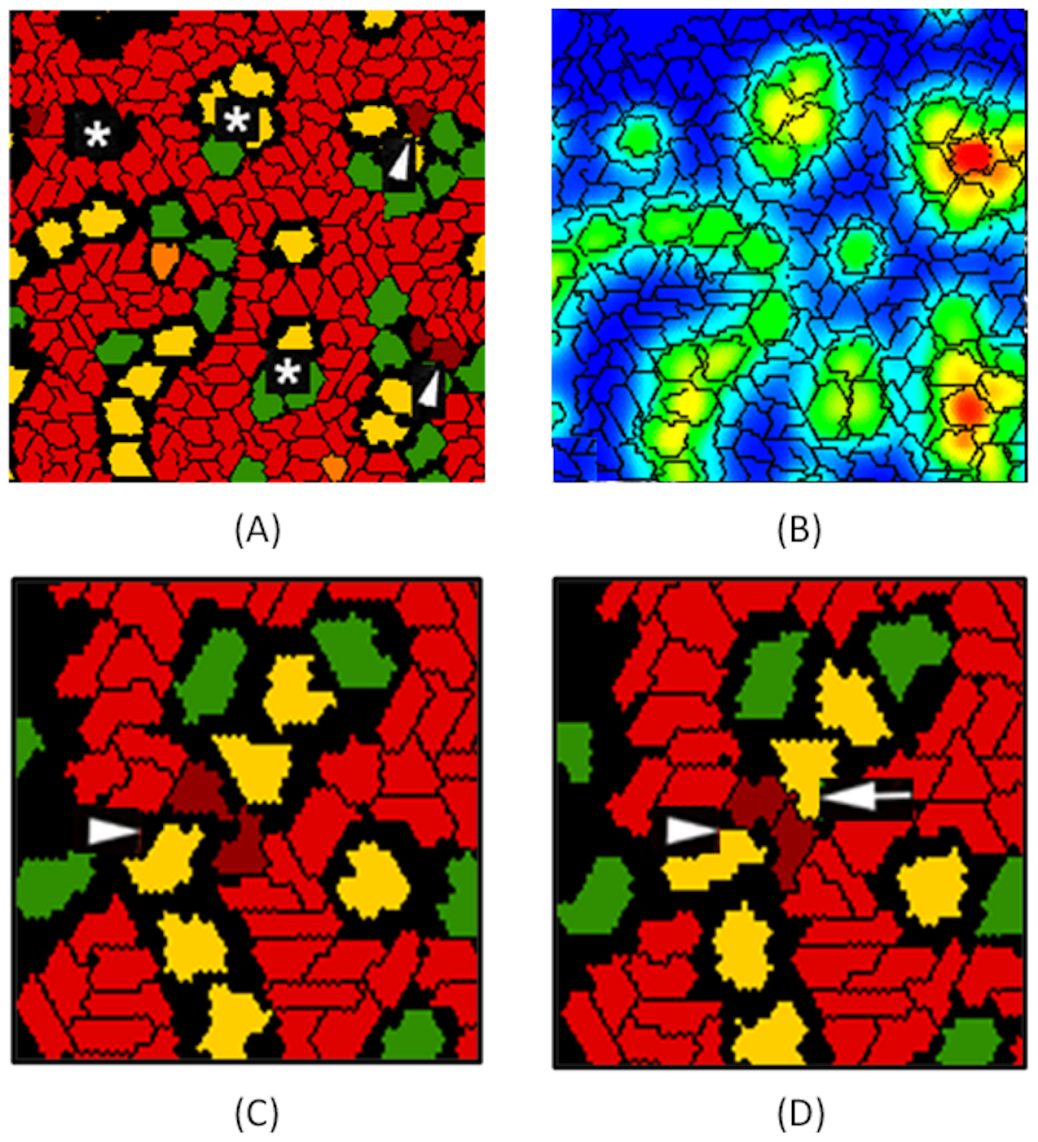
Phenotypic and emergent properties of simulated capillary plexus. (A,B) The hexagonal lacunae formed by the control vascular network model are marked by asterisks, and the arrowheads indicate angiogenic sprouts forming off the nascent vessels. In the computational model, these were seen in response to a local VEGF gradient (green to red color scale indicating low to high concentration). (C,D) Details of EC-tip cell interactions during vascular network formation. Mural cells (green) and inflammatory cells (yellow) interact with endothelial tip cells (dark red) to facilitate bridging between nascent vessel sprouts (arrowheads); endothelial stalk cells (light red) follow and later in time (D) the connection is formed. Macrophage-tip cell bridging (arrow) is an emergent feature of the *in silico* model and mimics events described during embryogenesis *in vivo* [31].

The emergent capillary network is locally regulated by molecular signals in concentration fields that are determined by different rates of diffusion, decay, secretion and/or uptake, detailed in the Methods section and depicted graphically in Figure 2. These signals may be freely diffusive (e.g., VEGF121 and sVEGFR1) or tightly bound to the ECM (VEGF165) or cell membrane (TIE2). Some signals depend on the presence of a specific cell-type and are thus transitory in nature. Protease expression in EC-tip cells is, for example, more prominent earlier in the simulation when a significant amount of exploratory behavior and vessel sprouting occurs. The visual outcome of the simulation (Figure 3 A,B) recapitulated formation of the primitive capillary plexus in an early embryo [13]. These features included quasi-hexagonal lacunae formed by the vascular network, uniform vessel thickness of 2–3 cells, regular branch points, and angiogenic sprouts forming off the nascent vessels in response to local growth factor gradients.

### Emergent Properties

During nascent angiogenic sprout formation in the ABM, EC-tip cells continually explore their environment via filopodial extensions. This orients a tip cell chemotactically along a relevant growth factor gradient, such as VEGF. In contrast, EC-stalk cells follow and proliferate behind the exploratory EC-tip cells. In the computational model, EC-tip cells responded chemotactically to gradients of VEGF165, VEGF121 and CCL2 (see Table 4). These molecules, plus the anti-angiogenic chemokine CXCL10, are secreted by ICs; VEGF165 is also liberated from the ECM by secreted proteases. The CCL2 chemokine also has a chemotactic effect on IC. An interplay between concentration fields of these signal molecules and the differential adhesion strengths between cell types facilitated cell-cell interactions as the endothelial network forms (Fig. 3C,D). This selective bridging of EC-tip cells, encouraged by macrophages, brought spatially seperated EC-tip cells into juxtaposition. This emergent property of the multicellular model mimics a ‘bridging phenomenon’ observed *in vivo* in mice during retinal angiogenesis, and in zebrafish vascular development [31].

### ToxCast Assay Results

**Figure 4:**
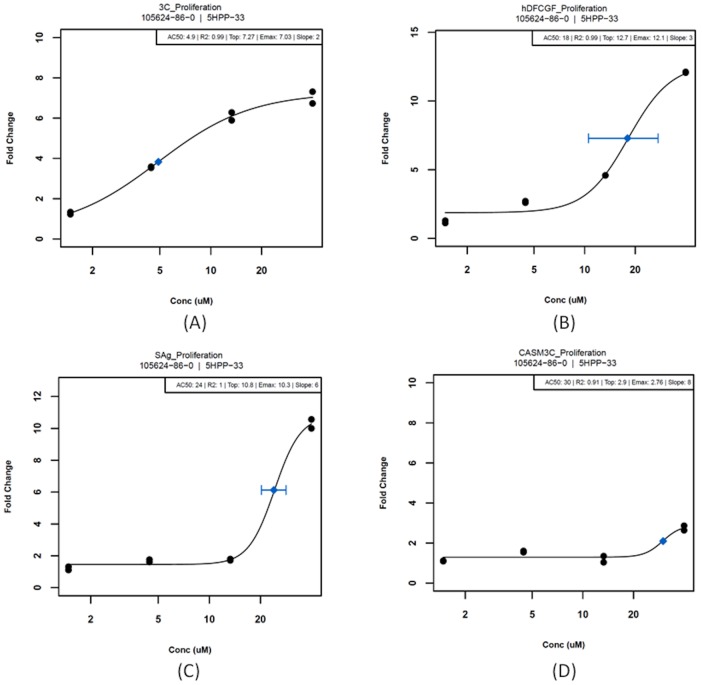
5HPP-33 results in the BioMAP system measuring fold change levels for inhibition of proliferation in (a) endothelial cells, (b) fibroblasts, (c) a co-culture of endothelial cells and peripheral blood mononuclear cells and (d) smooth muscle cells. Blue diamonds represent half-maximal activity concentrations (AC_50_) with standard error bars.

To evaluate the performance of the cell ABM for predictive toxicology, the anti-angiogenic reference compound, 5HPP-33 was tested across a series of HTS and HCS assay systems. Results in the BioMAP system showed numerous targets relevant to inflammatory and vascular pathways (Table 5). The lowest effective concentration (LEC) is the *in vitro* test concentration at which a response was observed that was significantly different (P≤0.01) from control. These are reported alongside the AC_50_ values, where available, based on the concentration response data. A maximum response (E_max_) of ≥2-fold change was required to fit a curve and generate an AC_50_. Examples of the concentration-response curves (for inhibition of proliferation in several BioMAP vascular cell systems) are shown in Figure 4. 5HPP-33 invoked EC-specific inhibition of proliferation at low concentrations (here defined as LEC ≤ 5 µM) and affected other cell types to varying degrees at higher test concentrations (here defined as 5 µM ≤ LEC ≤ 40 µM). There were a number of other targets affected by 5HPP-33 across the assay systems, including some that may be relevant to vascular development but have not yet been incorporated into the computational model. The full data set for 5HPP-33 is provided in Supplemental Table S3.

**Table 5:** 5HPP-33 results in the BioMAP system measuring fold change levels for downregulation/upregulation of protein levels in human primary cells.

Assay	Gene	AC_50_	LEC	E_max_	Model Parameter
BSK_3C_Proliferation_down	NA	4.9	1.5	7.03	Δ*V_t_(EC_s_)*
BSK_LPS_VCAM1_down	VCAM1	17	4.4	3.55	*J(IC:EC_s_), J(MC:EC_s_), T(EC_s_)*
BSK_hDFCGF_Proliferation_down	NA	18	4.4	12.1	*ΔV_t_(IC)*
BSK_hDFCGF_VCAM1_down	VCAM1	19	4.4	4.4	*J(IC:EC_s_)*
BSK_SAg_Proliferation_down	NA	24	4.4	10.3	*ΔV_t_(EC_s_)*
BSK_CASM3C_Proliferation_down	NA	30	4.4	2.76	Δ*V_t_(MC)*
BSK_4H_SRB_down	NA	NA	13	1.44	*P_apop_(EC)*
BSK_3C_SRB_down	NA	16	13	2.09	*P_apop_(EC)*
BSK_hDFCGF_PAI1_down	SERPINE1	17	13	3.53	*T(MC)*
BSK_hDFCGF_IP10_down	CXCL10	19	13	5.47	*S_Cx:IC_*
BSK_CASM3C_VCAM1_down	VCAM1	NA	13	1.55	*J(MC:EC_s_)*
BSK_3C_uPAR_down	PLAUR	20	13	2.6	*T(EC_t_), T(EC_s_)*
BSK_3C_VCAM1_down	VCAM1	20	13	2.03	*J(MC:EC_s_)*
BSK_4H_uPAR_down	PLAUR	23	13	2.33	*T(EC_t_), T(EC_s_)*
BSK_KF3CT_IP10_down	CXCL10	23	13	7.16	*S_Cx:IC_*
BSK_LPS_SRB_down	NA	NA	13	1.87	*P_apop_(EC)*
BSK_SAg_PBMCCytotoxicity_up	NA	NA	13	1.14	*P_apop_(IC)*
BSK_SAg_SRB_down	NA	NA	13	1.9	*P_apop_(EC)*
BSK_4H_MCP1_down	CCL2	NA	40	1.43	*S_C:EC_*
BSK_4H_VEGFRII_down	KDR	NA	40	1.45	*J(EC_s_:EC_s_), J(EC_t_:EC_s_), C_v:thresh_, S_Vf:Ecs_*
BSK_4H_VCAM1_down	VCAM1	24	40	2.2	*J(MC:EC_s_)*
BSK_SAg_MCP1_down	CCL2	28	40	4.61	*S_C:EC, IC_*
BSK_LPS_MCP1_down	CCL2	29	40	2.69	*S_C:EC, IC_*

The corresponding gene targets and computational model parameters are shown. SRB represents total protein levels, and is taken as a cytotoxicity measure. AC_50_: half-maximal activity concentration, LEC: Lowest Effective Concentration, E_max_: Maximal response.

### Anti-Estrogenic Activity of 5HPP-33

**Figure 5:**
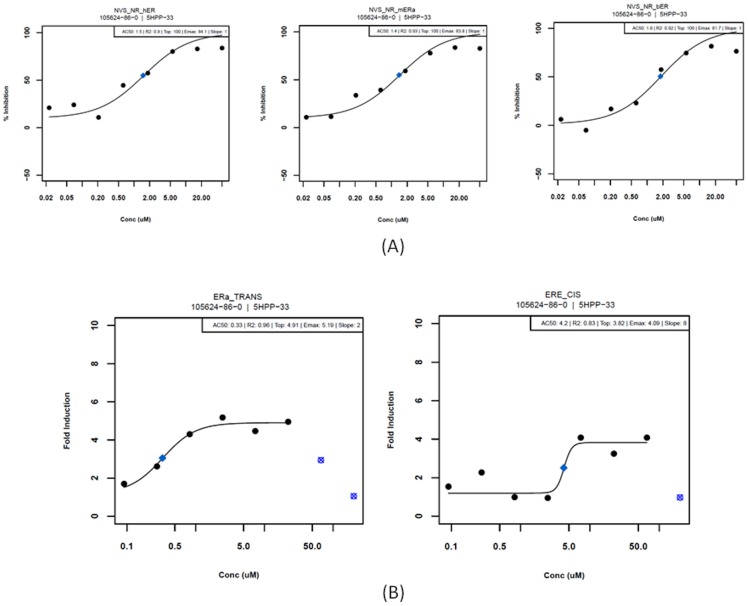
Modulation of the estrogen receptor (ER) by 5HPP-33. (A) 5HPP-33 results exhibiting binding of the ER in human (NR_hER), mouse (NR_mERa) and bovine (NR_bER) in NovaScreen system. Blue diamonds represent half-maximal activity concentrations (AC50). (B) 5HPP-33 targeted estrogen receptor alpha transcription factor activity (ERa_TRANS) and the cis-regulatory estrogen response element (ERE_CIS) construct in the Attagene system. Blue diamonds represent half-maximal activity concentrations (AC_50_). Overt cytotoxicity is seen at higher test concentrations; these points are flagged as outliers so that they do not contribute to the reported AC_50_ estimate.

An unexpected result of the ToxCast HTS screening was the strong activity exhibited by 5HPP-33 against the estrogen receptor (ER). When tested in NovaScreen cell-free biochemical assays, 5HPP-33 exhibited concentration-dependent binding to estrogen receptors from multiple species. The AC_50_ was 1.4 µM in mouse ER-alpha, 1.5 µM in human ER, and 1.8 µM in bovine ER binding assays (Fig. 5A). In the Attagene reporter assays, 5HPP-33 affected ER-alpha transcription factor activity (ERa_TRANS) with an AC_50_ of 0.39 µM and the cis-regulatory estrogen response element (ERE_CIS) construct with an AC_50_ of 4.4 µM (Fig. 5B). The effect of binding to the estrogen receptor is represented implicitly in the model via a surrogate effect on VEGF secretion (*S_Vf:ECs_*), based on the known transcriptional relationship between ER and VEGF [61].

### Adverse Outcome Pathway (AOP) for 5HPP-33

**Figure 6:**
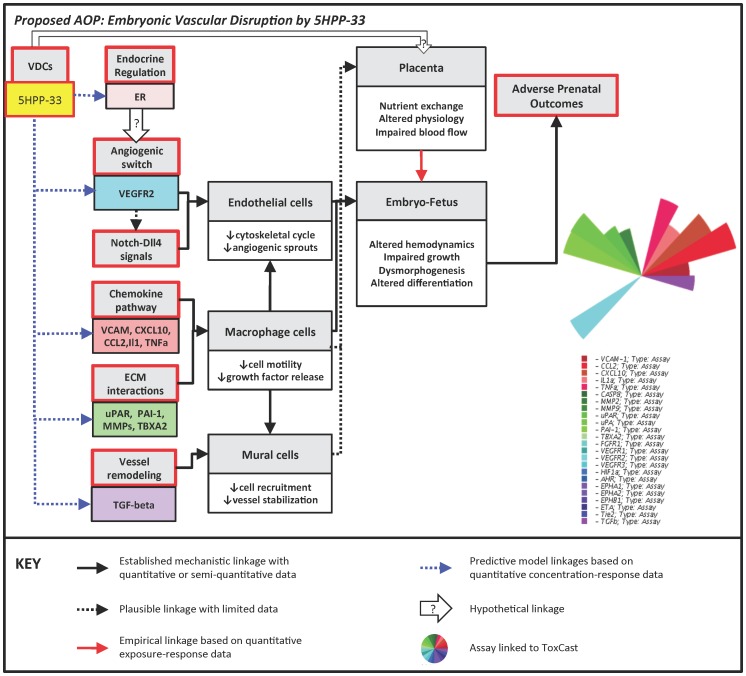
Proposed Adverse Outcome Pathway (AOP) for embryonic vascular disruption by 5HPP-33. Boxes on the left represent molecular initiating events, leading to adverse prenatal outcomes via the associated pathways. The hypothetical linkage between the estrogen receptor (ER) and the angiogenic switch is based on the ToxCast assay results and the known transcriptional relationship between ER and VEGF. In the graphic at right, the colored slices corresponding to each assay are scaled so that more potent (i.e. lower AC_50_) results extend further from the origin.

An AOP framework anchoring molecular initiating events (MIEs) to adverse outcomes at the individual or the population level was previously developed for embryonic vascular disruption leading to developmental toxicity [8]. Based on that framework, the putative AOP for embryonic vascular disruption by 5HPP-33 is shown in Figure 6. Examining the ToxCast HTS assay results represented in the computational model, 5HPP-33 affected the angiogenic switch via down-regulation of the VEGFR2 receptor and the chemokine pathway via downregulation of CCL2, CXCL10, IL1a, TNFa and other inflammatory signaling molecules. It also targeted vessel remodeling via down-regulation of TGFβ and ECM matrix interactions through a variety of MMPs and PAS targets, including uPAR, uPA and PAI-1. An additional direct target in the biochemical cell-free assay platform was thromboxane A2, which in addition to its' role in clot formation, regulates ECM gene and protein expression and migratory capabilities of various cell types [62], [63]. Based on the estrogenic results across multiple assay types and platforms, we would also hypothesize that 5HPP-33 influences VEGF transcription and angiogenic growth factor signaling via an endocrine-regulated pathway.

### 5HPP-33 Simulations

The ToxCast data for 5HPP-33 were translated into model parameter perturbations, as shown in Table 5 and detailed in the Methods section, based on the LECs for each target. Where possible, fold changes in protein levels from the concentration response curves (Supplemental S3) were translated directly into parameter fold changes. For example, a 2-fold decrease in CCL2 levels resulted in a 2-fold decrease in the CCL2 secretion rate parameter for each corresponding cell type. Changes in proliferation were equivalently applied as adjustments to the change in target volume of the respective cell types. For example, at 40 µM 5HPP-33 caused ∼7-fold inhibition of endothelial cell proliferation (Fig. 4, 3C system) vs. ∼3-fold inhibition of smooth muscle cells (Fig. 4, CASM3C system) at the same concentration; equivalent decreases were directly applied to Δ*V_t_(EC_s_)* and Δ*V_t_(MC)*, respectively, as shown in Table 5. Others were not as straightforward, such as VCAM1, which influences cell-cell adhesion and transendothelial leukocyte migration and therefore is translated into contact energies between cells and cell type-specific motility, dimensionless computational parameters. In such cases there was no way to directly translate fold changes so a heuristic was applied in the form of incremental decreases in parameters. A 2-fold decrease in VCAM1 caused a drop in the contact energy between EC and IC from −5 to −4, for example. The proposed decrease in VEGF secretion by ECs due to ER-binding and VEGFR2 inhibition (a 10-fold drop in EC-specific VEGF secretion, without affecting secretion by the other cell types) was implemented at the highest test concentration of 40 µM. The XML and Python configuration files used to parameterize and run each simulation are included in the Supplemental Material, and the parameters adjusted to mimic 5HPP-33 exposure were commented at the relevant places in the code.

**Figure 7:**
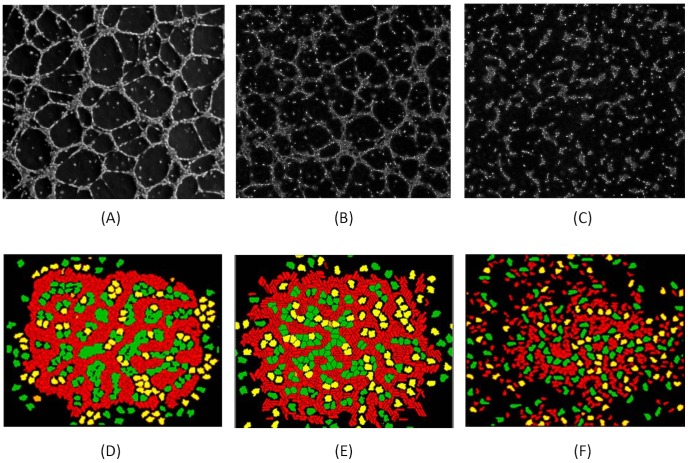
Consequences of 5HPP-33 exposure on vasculogenesis: phase-contrast photomicrographs of human umbilical vein endothelial cells (HUVEC) exposed to 5HPP-33 *in vitro* (A–C); previously unpublished HUVEC images were graciously donated by Prof. Hashimoto [56] and show monolayers plated at 5.0×10^5^ cells/well grown in DMEM containing angiogenic growth factors (hEGF, VEGF, hFGF-B, and R3-IGF-1) and treated with test compounds (0.5% DMSO vehicle control, 3 µM 5HPP-33, or 30 µM 5HPP-33) for 6 hours. Images are compared to *in silico* results predicted by the cell-ABM simulation with ToxCast HTS data after 10,000 MCS (D–F). (A,D) Control (DMSO vehicle): demonstrates the polygonal organization of endothelial cells (arrows) *in silico* (A) or 6 hr HUVEC culture (0.5% DMSO); bar = 100-µm (D). (B,E) Low-concentration 5HPP-33: simulation of ToxCast HTS data for features altered at or below 4.44 µM 5HPP-33 (B); a slightly less connected endothelial network is also observed following exposure to 3 µM 5HPP-33 (E). (C,F) High-concentration 5HPP-33: simulation of ToxCast HTS data for features altered at or below 40 µM 5HPP-33 (C); dispersion and inhibition of vessel formation is also observed following exposure to 30 µM 5HPP-33 (F).

The simulations of 5HPP-33 exposure during early embryonic vascular patterning were compared to similar test concentrations with HUVEC cultures stimulated to undergo vasculogenesis [56], to qualitatively assess the degree of vascular disruption and cellular pathophysiology. The experimental images were taken after 6 hours of chemical exposure (0.5% DMSO vehicle control, 3 µM 5HPP-33, or 30 µM 5HPP-33), a similar time scale to the simulations (10,000 MCS or ∼3 hours). Although the time points are not identical, and the computational model includes additional cell types (IC and MC), the EC patterning (red cells) may be compared with HUVEC vascular network formation in order to identify similar features. The normal (control) simulation showed typical plexus formation and patterning, with a high degree of connectivity and regular branching (Fig. 7A, D) similar to what was observed *in vitro*. The low concentrations (3 µM *in vitro*, compared to 4.44 µM *in silico*) showed partial disruption of plexus formation (Fig. 7B, E) that was more evident in the experimental images than the *in silico* results. There were isolated segments that form vessel networks but with a slightly lower degree of connectivity, and cellular clustering was observed in both cases. The high concentrations (30 µM *in vitro*, compared to 40 µM *in silico*) showed little to no vessel formation and a high occurrence of cellular clustering (Fig. 7C, F). The concordance between the simulated results and the experimental images suggests that the cell ABM had sufficient complexity and detail to learn new information about an important biological response.

### Quantitative Analysis with AngioTool

**Figure 8:**
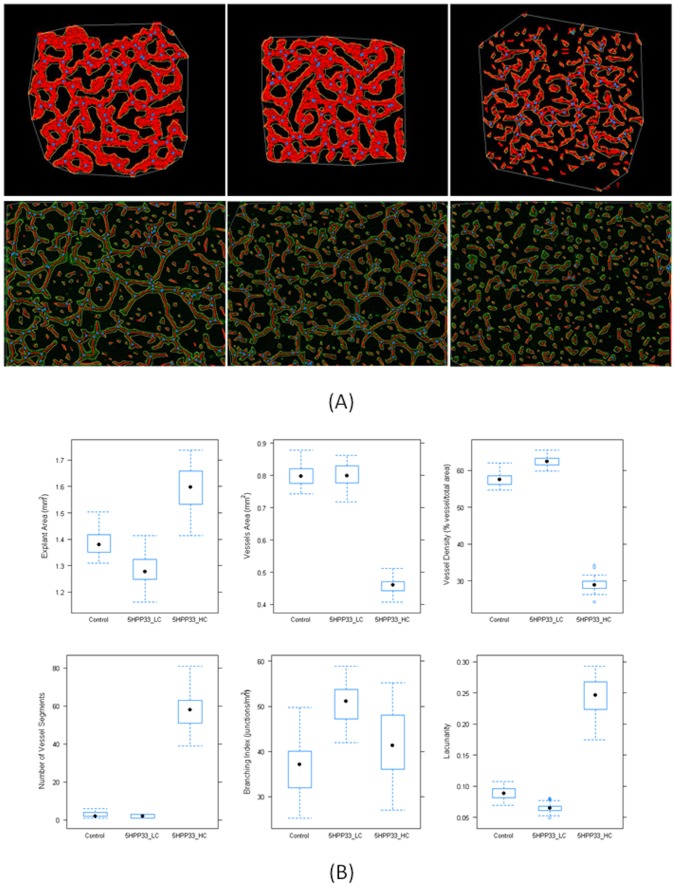
Quantitative analysis of 5HPP-33 exposure on simulated vascular plexus formation. (A) Upper panel shows representative images of AngioTool analysis performed on control, 4.44 µM (5HPP-33 LC) and 40 µM (5HPP-33 HC) simulation outputs. Lower panel shows AngioTool analysis performed on donated experimental images for control, 3 µM 5HPP-33 and 30 µM 5HPP-33 exposure conditions. (B) Graphical representation of explant area, vessels area, vessels percentage area, number of vessel segments, branching index, and lacunarity values for the control model, 4.44 µM (5HPP-33 LC) and 40 µM (5HPP-33 HC). N = 30 simulations for each exposure scenario.

Vascular network images generated from multiple simulations (n = 30) for each exposure scenario (control, 5HPP-33_LC (4.44 µM), and 5HPP-33_HC (40 µM)) were analyzed using the automated image processing software AngioTool (v0.5a). This tool was originally designed for use on experimental images such as allantois explants [60] and was adapted here for use with the *in silico* outputs from the cell ABM. The images were “stained” for ECs such that the simulated cell type colors were adjusted so that non-ECs appeared black to facilitate automated image processing. The first panel of Fig. 8A shows representative simulation outputs after they have been “stained” for ECs *in silico*, segmented and analyzed by AngioTool. Figure 8B shows a graphical representation of the distribution of various quantitative metrics of angiogenesis for 30 simulations in each exposure scenario (control, 5HPP-33_LC, and 5HPP-33_HC). The coefficient of variation (CV) in almost all cases was <10%, except in the case of branching index for all three conditions and lacunarity and number of vessel segments at the simulated high test concentration. The total explant area showed a significant decrease (p<0.0001) between the control model and the 5HPP33_LC, while the area occupied by the vessels was equivalent. This could be due to mild inhibition of proliferation of multiple cell types at the low test concentration, resulting in slightly stunted angiogenic outgrowth. The total explant area was increased for 5HPP33_HC, due to inhibition of cellular adhesion molecules and complete lack of vascular organization, as is evidenced by the large decrease in vessel density from ∼58% (control) to ∼29% (5HPP33_HC, p<<0.0001). Both 5HPP33_LC and the control scenario usually resulted in a fully developed and interconnected plexus (one vessel “segment”), whereas the number of vessel segments was significantly increased (p<<0.0001) to >50 after simulated exposure to the high test concentration. The lacunarity, an index for vascular structural nonuniformity, was shown to increase significantly at the high concentration, consistent with what has been observed in the case of other known VDCs [60], [64].

AngioTool analysis was also performed on representative experimental HUVEC images (Fig. 8A, lower panel) for control (DMSO vehicle), 3 µM, and 30 µM 5HPP-33 exposure conditions. Because the computational model was not parameterized to match these experimental conditions and contains additional cell types, measured values such as explant area or branching index would be expected to differ. However, parameters such as lacunarity and vessel density show a similar concentration response trend, where the lacunarity was equivalent in the control and the 3 µM case (0.16 and 0.17, respectively), and increased after exposure to 30 µM to 0.27, similar to what was predicted *in silico*. The vessel density also changed very little after exposure to the low test concentration, going from 32% in the control image to 30% in the 3 µM image, while it dropped to 20% in the 30 µM case. While the vessel density was estimated to be higher in the computational model (∼60% for control and 5HPP33_LC), the concentration response trend was predicted where the vessel density remained almost unchanged at the low concentration and showed a strong decrease at the high concentration. The full set of AngioTool image data (computational and experimental) and statistical analyses are provided as Supplemental Datasets S1–S4 and Supplemental Table S4.

## Discussion

Results from this study show that a cellular ABM with sufficient molecular complexity and mathematical detail can: (a) effectively simulate early embryonic vascular development including emergent properties such as macrophage bridging [13], [31]; (b) recapitulate the topology of a functional angiogenesis assay *in vitro* [56]; (c) incorporate HTS data to quantitatively predict the higher-order effects on vascular network formation [12]; and (d) simulate key events from molecular perturbations to tissue disruption in an Adverse Outcome Pathway (AOP) for embryonic vascular disruption [8]. Taken together, these results for the first time demonstrate the translation of a mathematical model into predicted biological responses utilizing computer simulation and *in vitro* HTS data.

The computational model simulated here includes a number of critical cell types and molecular signals needed for vasculogenesis and angiogenesis. Other studies have modeled vascular network formation using a cell agent-based strategy although have excluded control from the range of molecular signals possible in an embryological system. For example, previous models typically focused on one or two cell types (ECs, tumor cells), detailed analysis of a specific behavior (elongation, ECM interaction, tip cell selection) and/or the influence of one major growth factor (VEGF) via either paracrine or autocrine signaling [14]–[16], [18], [21], [65]. There are significant advantages and important insights gained from concentrating on a small number of cell types/signals/behaviors; however, for our purposes we strove to achieve a balance between sufficient degrees of biological complexity and simplifying assumptions that would allow for adequate recapitulation of embryonic vascular biology and subsequent prediction of chemical perturbation. This model expands upon a number of established models of plexus formation and incorporates the lessons learned and insights gained from previous approaches [14], [15], [16], [18]. Previous work done also provided excellent starting points for parameter range-finding and sensitivity analysis, and assisted in choosing values that would minimize computational artifacts. The current model does not assume exclusively paracrine or autocrine signaling, but rather a combination of both, to best mimic an *in vivo* scenario. In addition, rather than one growth factor driving the patterning of a single cell type, here there were several molecular signals and cell types interacting to provide both pro- and anti-angiogenic cues. The net outcome is a stable capillary plexus that phenocopies what can be observed in the early embryo, but with subtle emergent features such as nascent vessel stabilization/remodeling and macrophage-tip cell bridging [31], [32] that may be important to the timing and patterning of embryonic vascular development, and to the genetic or environmental determinants of susceptibility.

Genetic studies have shown that perturbing vascular signals can lead to varying degrees of adverse consequences, ranging from congenital angiodysplasia to fetal malformations and embryolethality. Furthermore, evidence for chemical disruption of vascular developmental processes is available for thalidomide, estrogens, endothelins, dioxin, retinoids, cigarette smoke, and metals among other compounds. Exposure to these ‘Vascular Disruptor Compounds’ (VDCs) has been shown to cause a wide range of developmental adverse outcomes (phocomelia, cleft palate, neural tube defects, preeclampsia, embryolethality, fetal weight reduction, etc.) in a variety of *in vivo* animal models and human epidemiological data [8]. This provides compelling evidence for the value of computational models and simulations to predict effects of chemical exposure; however, their value should not be judged solely on the biological complexity and mathematical detail, but on what can be learned from them. Models that work, and that work for the right reason, offer the potential use as an *in silico* platform in predictive toxicology for assessing the potential consequences of drug or chemical exposure to embryonic vascular development. First-generation predictive models built from ToxCast HTS data and linked to apical *in vivo* endpoints include chronic liver cancer in rodents [66], reproductive toxicity in rats [67], prenatal developmental toxicity in rats and rabbits [68], and multi-organ carcinogenesis [69]. We previously performed a biologically-based analysis of the data assisted by semi-automatic knowledgebase curation that revealed a number of angiogenic targets in inflammatory chemokine signaling, the VEGF pathway, and the PAS were strongly perturbed by some environmental compounds with positive correlations to developmental effects. This led to the development of a predictive model for ‘putative VDCs’ (pVDCs) based on ToxCast Phase I HTS data [12] and expansion of this model into a conceptual AOP framework for embryonic vascular disruption [8]. The cell types and molecular targets identified as critical to early embryonic vascular patterning were incorporated into the cellular ABM, and comprised a sufficient level of biological complexity to reproduce normal capillary formation and chemical disruption.

Here, we followed this conceptual framework and developed an AOP for embryonic vascular disruption by the anti-angiogenic thalidomide analogue 5HPP-33 based on the ToxCast HTS data for targets in angiogenic growth factor signaling, chemokine pathways, ECM interactions, and vessel remodeling, and simulated it using the cellular ABM. The simulation predicted an initial effect at the low test concentration on EC proliferation, motility and adhesion. The multi-faceted impact on EC resulted in a more rigid cell shape, as shown in Figure 7B, which is consistent with another proposed mechanism of 5HPP-33, microtubule stabilization [70]. At higher test concentrations, there were additional targets controlling ECM interactions, growth factor and chemokine signaling, and proliferation of other cell types. The presence of MC and IC in the simulation appeared to partially rescue early vascular cord formation, especially in the areas of high cell density, but this occurred over a much slower time scale than in the control model. Following image analysis by AngioTool, we were able to assess the variability in our model and make quantitative predictions showing significant changes in metrics of angiogenic disruption such as explant area, vessel density, number of segments and lacunarity, following exposure to increasing concentrations of 5HPP-33. The branching index, measuring the number of junctions per unit area, did not show a significant concentration dependent response and was also the metric that exhibited the most variability. This may be because the image segmentation skeleton was not properly optimized for each exposure scenario (the same analysis parameters were used for each case to minimize sources of uncertainty) and the number of junctions was overestimated for the 5HPP33_HC outputs. Interestingly, at the low concentration the model predicted a slight decrease in lacunarity and increase in branching index (due to a similar number of junctions over a smaller area), that is likely due to the inhibition of proliferation of various cell types observed in the ToxCast assays, but may also correlate with the mechanism of microtubule stabilization. This demonstrates the need for further targeted assays providing insight on how biological information flows from one cellular property to another, and how it is influenced by local factors such as cell density and heterogeneity.

The disruptive effects of 5HPP-33 on EC proliferation and vascular network formation may also occur in part via estrogen receptor (ER) dependent signaling and a decrease in VEGF transcription. The AC_50_ values for the cell-free, biochemical ToxCast assays measuring ER binding and gene transcription were less than or equal to the concentrations at which significant changes in protein levels were observed. Hormonally controlled vascular changes are known to play a key role in endometrial development and blastocyst implantation, and estrogen-dependent VEGF is known to be a central regulator of uterine vasculature permeability, placentation and angiogenesis during the peri- and post-implantation period [71]. While there is a clear potential for adverse developmental outcomes that may follow from endocrine-mediated disruption of angiogenesis, the mechanisms are not yet known. Estradiol has been shown to promote EC migration, proliferation and inhibition of apoptosis through ERα-mediated pathways, while the estrogen metabolite 2-methoxyoestradiol has demonstrated potent anti-angiogenic properties mediated by cytoskeletal actions and an increase in EC apoptosis, and several non-steroidal anti-estrogens (clomiphene, nafoxidine, tamoxifen); pure ER antagonists inhibited angiogenesis in the chick chorioallantoic assay [61]. Estrogenic compounds have been shown to modulate VEGF transcription and secretion via ERα and ERβ in both a positive and negative direction in various cancer cell lines [72]–[74], as well as in mesenchymal stem cells and murine embryonic lung cells [75], [76]. Estrogens and selective ER modulators have been shown to inhibit vascular smooth muscle cell proliferation and endothelial VCAM1 expression [77], [78]. In the present HTS study data, at test concentrations ≤5 µM, 5HPP-33 down-regulated VCAM1 expression in ECs and ICs, representing a possible downstream target of this partial/selective ER agonist. A postulated AOP for embryonic vascular disruption by 5HPP-33 therefore includes the molecular initiating event of ER binding leading to inhibition of growth factors and cell adhesion molecules.

The computational model of early embryonic vascular development includes the key cell types and molecular signals that cooperate to promote initial capillary plexus formation. There are a number of extensions to the model under consideration, including the incorporation of intracellular signaling networks [79] and additional components such as the ER, identified here as a novel target of the thalidomide compound 5HPP-33. Another limitation of the model that remains to be addressed is the lack of blood flow, which can be incorporated when the model is expanded into three dimensions. A comprehensive sensitivity analysis of every parameter was outside the scope of the present study; however, the vast body of vascular modeling literature that exists informed parameter range estimates for secretion rates, chemotaxis, motility and so forth. For stochastic cell-level behaviors such as cell growth and apoptosis, the present study performed parameter sweeps to optimize cell number and VEGF concentration for model development. An examination of varying patterns of chemical perturbations in future work will shed more light on which molecular targets, both uniquely and in combination, are predicted to have the most impact on plexus formation. However, while the current 2D model makes a number of simplifying assumptions, the degree of biological complexity was sufficient to reproduce key morphological features and emergent behaviors during vascular development. This *in silico* model also demonstrates a novel approach using *in vitro* assay data on a cellular and molecular scale to accurately predict qualitative phenotypic changes on a tissue scale with increasing concentrations of an anti-angiogenic compound. The quantitative outputs from the AngioTool analysis show a high degree of reproducibility across multiple simulations for each scenario, and, when combined with the qualitative comparisons to experimental results, provide new biological insight and quantifiable predictions relating molecular and cellular changes to disruption of vascular development. Although we have framed the current vascular model in the context of embryonic development, there is significant overlap between developmental and pathological angiogenic signaling [2], and such a model could be potentially useful to a wide range of applications in wound healing and tumor angiogenesis.

Traditional *in vivo* animal testing, usually at high test doses, is low-throughput and costly both in terms of financial and animal resources. These restrictions result in a relatively low number of compounds that have sufficient *in vivo* data to assess the potential for adverse effects on human development. Toxicity testing in the 21^st^ century is moving toward using HTS assays to rapidly test thousands of chemicals against hundreds of molecular targets and biological pathways, to provide mechanistic information on chemical effects in human cells and small model organisms, and to construct predictive and mechanistic models [80], [81]. Virtual tissue simulations may someday serve as a surrogate to animal testing by providing *in silico* testing platforms based on computational systems biology [82]. In conjunction with ToxCast HTS data, the model presented here has the potential to predict toxic effects caused by exposure to anti-angiogenic compounds, comparable to *in vitro* and *in vivo* angiogenesis assays.

## Supporting Information

Dataset S1 Quantitative metrics of angiogenesis from AngioTool analysis of the output of the control model, n = 30 simulations.
(XLSX)

Dataset S2 Quantitative metrics of angiogenesis from AngioTool analysis of the output of the model of 5HPP-33 exposure, low concentration (4.44 µM), n = 30 simulations.
(XLSX)

Dataset S3 Quantitative metrics of angiogenesis from AngioTool analysis of the output of the model of 5HPP-33 exposure, high concentration (40 µM), n = 30 simulations.
(XLSX)

Dataset S4 Comparison of AngioTool metrics (mean, std. dev.) between the control model, 5HPP-33 low concentration, and 5HPP-33 high concentration.
(XLSX)

Software S1 XML and Python code for computational model of early embryonic vascular plexus formation. Files included for control model, 5HPP-33 low exposure of 4.44 µM (LD), and 5HPP-33 high exposure of 40 µM (HD) are labeled accordingly.
(ZIP)

Table S1 AngioKB.v1 (divided into parts A and B, due to file size) electronic library for blood vessel development and remodeling, built and curated semi-automatically from the open scientific literature.
(ZIP)

Table S2 Concentration response data for 5HPP-33, tested in 274 ToxCast assays across the Attagene, Novascreen, and Bioseek platforms.
(XLSX)

Table S3 Comparison of selected AngioTool metrics between the simulation outputs (control model, 5HPP-33 low concentration, and 5HPP-33 high concentration) and representative experimental images. Significance was calculated based on student's t-test p-values.
(XLSX)

Video S1 Control model of early embryonic vascular plexus formation over 10,000 MCS (∼3 hours). Red cells are endothelial cells, green cells are mural cells and yellow cells are inflammatory cells.
(GIF)

Video S2 Control model of early embryonic vascular plexus formation over 10,000 MCS, showing the cellular lattice and overlaid molecular signaling concentration fields.
(GIF)

Video S3 Control model of early embryonic vascular plexus formation over 10,000 MCS showing “in silico staining” of endothelial cells, where mural cells and inflammatory cells are present but colored black.
(GIF)
