# Supplementary figures and images for: A Computational Model Predicting Disruption of Blood Vessel Development
Source: PLoS Comput Biol. 2013 Apr 4;9(4):e1002996. doi: 10.1371/journal.pcbi.1002996 (PMC3616981; doi:10.1371/journal.pcbi.1002996)

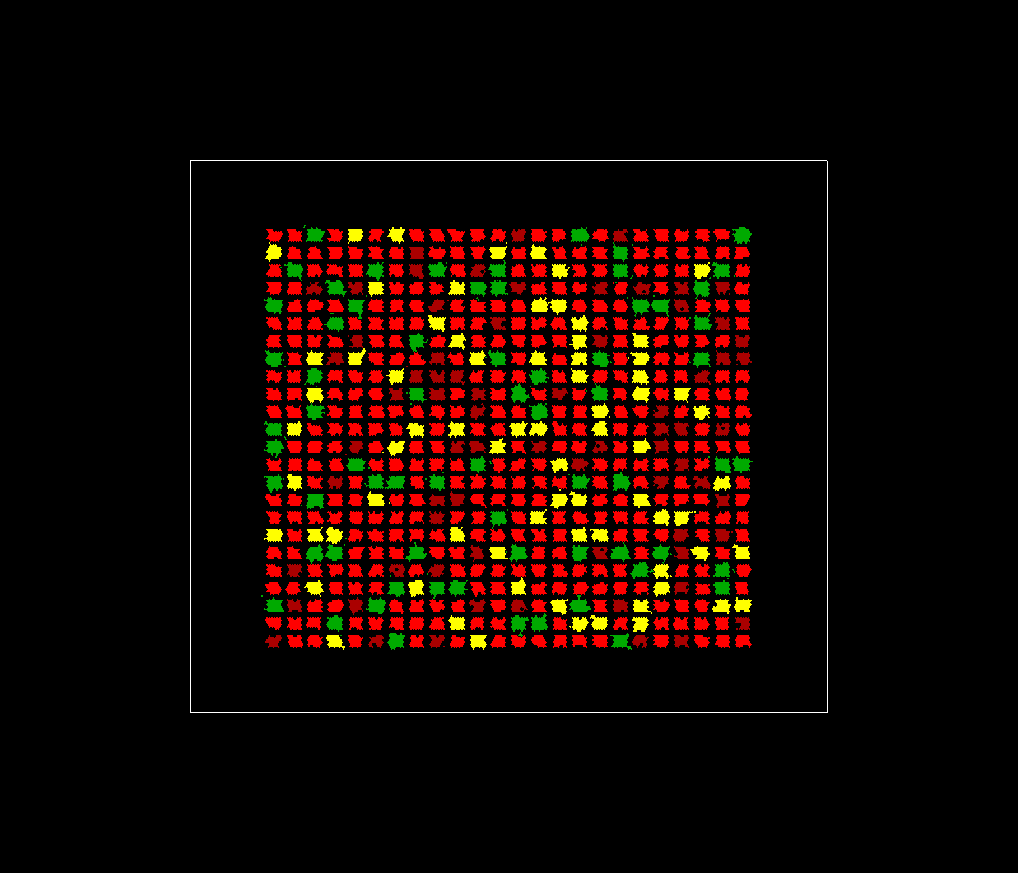

Supplement: Video S1 — Control model of early embryonic vascular plexus formation over 10,000 MCS (∼3 hours). Red cells are endothelial cells, green cells are mural cells and yellow cells are inflammatory cells. (GIF) [file pcbi.1002996.s009.gif]

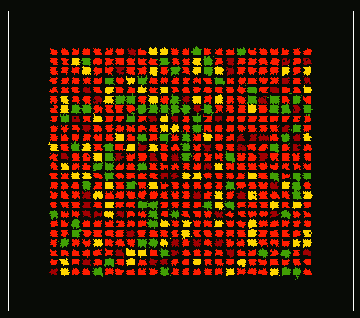

Supplement: Video S2 — Control model of early embryonic vascular plexus formation over 10,000 MCS, showing the cellular lattice and overlaid molecular signaling concentration fields. (GIF) [file pcbi.1002996.s010.gif]

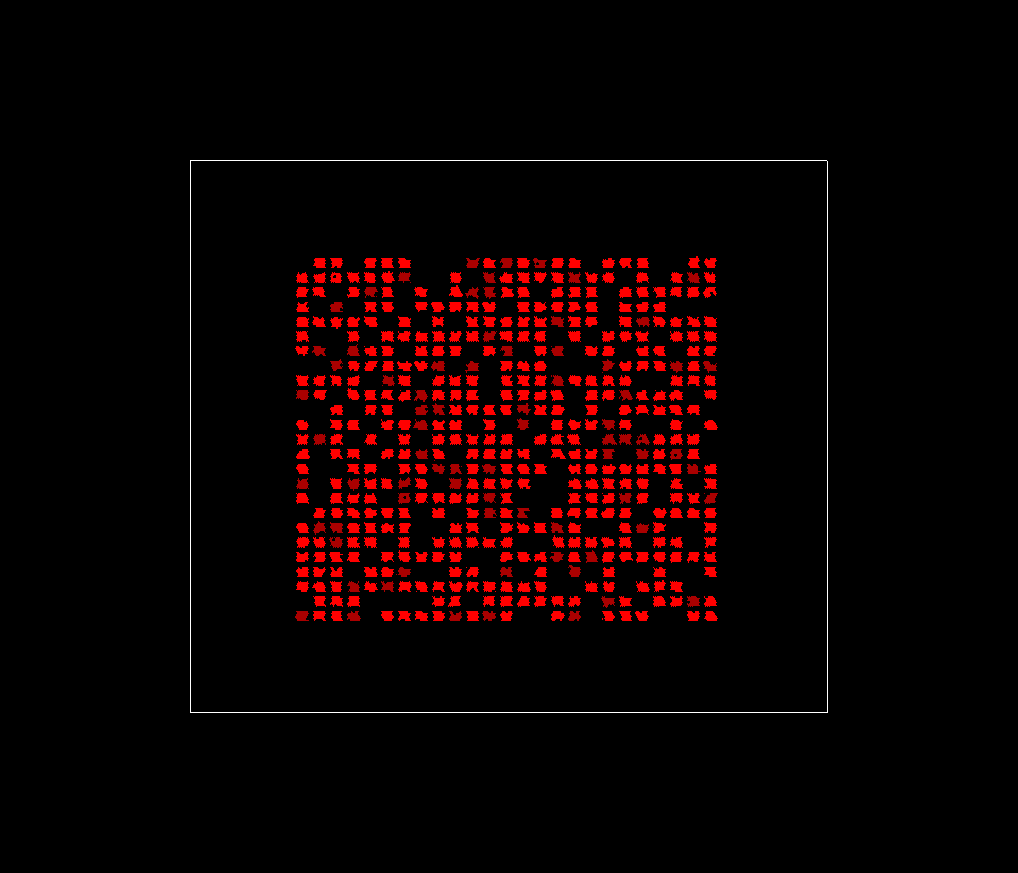

Supplement: Video S3 — Control model of early embryonic vascular plexus formation over 10,000 MCS showing “in silico staining” of endothelial cells, where mural cells and inflammatory cells are present but colored black. (GIF) [file pcbi.1002996.s011.gif]
